# Association of TNF-α, TNFRSF1A and TNFRSF1B Gene Polymorphisms with the Risk of Sporadic Breast Cancer in Northeast Chinese Han Women

**DOI:** 10.1371/journal.pone.0101138

**Published:** 2014-07-10

**Authors:** Fengyan Xu, Guiqin Zhou, Shaoli Han, Weiguang Yuan, Shuang Chen, Zhenkun Fu, Dalin Li, Hua Zhang, Dianjun Li, Da Pang

**Affiliations:** 1 Department of Anatomy, Harbin Medical University, Harbin, China; 2 Department of Immunology, Heilongjiang Provincial Key Laboratory for Infection and Immunity, Harbin Medical University, Harbin, China; 3 Institute of Cancer Prevention and Treatment, Harbin Medical University, Harbin, China; 4 Department of Surgery, the Third Affiliated Hospital of Harbin Medical University, Harbin, China; Kyung Hee University, Republic of Korea

## Abstract

**Background:**

The interaction of tumor necrosis factor-α (TNF-α) with its receptors: TNFRSF1A and TNFRSF1B is critical for the promotion of tumor growth, invasion and metastasis. To better understand the roles of single nucleotide polymorphisms (SNPs) in the TNF-α, TNFRSF1A and TNFRSF1B genes in the development of breast cancer, we explored the associations between SNPs in these three genes and breast cancer susceptibility in northeast Chinese Han women.

**Methodology/Principal Findings:**

This case-control study was conducted among 1016 breast cancer patients and 806 age-matched healthy controls. Seven SNPs in the TNF-α (rs1800629, rs361525), TNFRSF1A (rs767455, rs4149577 and rs1800693) and TNFRSF1B (rs1061622 and rs1061624) genes were genotyped by polymerase chain reaction-restriction fragment length polymorphism (PCR-RFLP) method. In TNFRSF1B, the rs1061622 GT genotype and the G allele conferred a reduced susceptibility to breast cancer (P = 0.000662, OR = 0.706, 95% CI: 0.578–0.863; P = 0.002, OR = 0.769, 95% CI; 0.654–0.905, respectively). Moreover, the AG genotype, the AA genotype and the A allele in rs1061624 conferred an increased risk of breast cancer (P = 0.007, OR = 1.470, 95% CI:1.112–1.943; P = 0.00109, OR = 1.405 95% CI:1.145–1.724; P = 0.001, OR = 1.248 95% CI:1.092–1.426, respectively). These two SNPs also had associations with breast cancer risk under the dominant model. In haplotype analysis, the CTA (rs767455 C-rs4149577 T-rs1800693 A) haplotype in TNFRSF1A and the TA (rs1061622 T-rs1061624 A) haplotype in TNFRSF1B had higher frequencies in breast cancer patients (P = 0.00324; P = 0.000370, respectively), but the frequency of GG (rs1061622 G-rs1061624 G) haplotype in TNFRSF1B was lower in breast cancer patients (P = 0.000251). The associations of the three haplotypes remained significant after correcting for multiple testing. In addition, significant associations were also observed between TNFRSF1A polymorphisms and lymph node metastasis, P53, estrogen receptor (ER) and progesterone receptor (PR) statuses.

**Conclusions:**

Our results suggest that rs1061622 and rs1061624 in TNFRSF1B may affect breast cancer risk, and SNPs in TNFRSF1A are associated with the clinical features of breast cancer.

## Introduction

Breast cancer is one of the major cancers affecting the mortality of women worldwide. The development of breast cancer has been shown to result from complex interactions between genetic and environmental factors. A single nucleotide polymorphism (SNP) is the most common genetic variation, and this variant attracts much attention because of its effect on biological function. Recently, accumulating evidence has shown that chronic inflammation in the vicinity of the tumor microenvironment can promote the growth as well as the progression of breast cancer [1], [2]. Therefore, SNPs in inflammation-related cytokine genes may be some of the influencing factors that affect breast cancer susceptibility.

TNF-α is an inflammatory cytokine mainly produced by activated macrophages and monocytes. TNF-α induces a cascade of other inflammatory cytokines and chemokines, and has been considered as one of the key mediators of inflammation. When expressed locally by immune cells, TNF-α has a therapeutic role in destroying tumor blood vessels and inducing the apoptosis and necrosis of tumor cells [3]. However, when chronically produced and inflammation persists in the tumor microenvironment, TNF-α can act as a tumor promoter by promoting DNA damage, enhancing pro-angiogenic functions, increasing the expression of matrix metalloproteinases (MMP) and endothelial adhesion molecules and inducing a milieu of growth-promoting hormone [4]–[6]. Furthermore, the chronic expression of TNF-α in breast tumors has been demonstrated to be correlated with lymph node involvement, suggesting the role of TNF-α in enhancing tumor cell metastasis [7]. Moreover, it has been observed that breast cancer patients with elevated levels of TNF-α in the circulation have a poor prognosis. TNF-α thus constitutes a useful biomarker in cancers [8], [9].

TNF-α executes its multiple biologic functions by binding to two distinct receptors, TNFRSF1A (TNFRI; p55; CD120*a*) and TNFRSF1B (TNFRII; p75; CD120b). The interactions of three genes are involved in inducing the production of other cytokines and promoting tumor growth through the activation of NF-KappaB [10], [11]. The abnormal expression of these three genes is involved in the pathogenesis and treatment outcomes of various malignant tumors, including breast cancer [12], [13]. Moreover, in breast cancer cell lines, blocking TNFRSF1A or TNFRSF1B with specific antibodies impairs tumor survival signaling and the biological function of TNF-α [11]. These results indicate that TNFRSF1A and TNFRSF1B also play important roles in tumor cell proliferation, invasion and metastasis.

Due to the important role of TNF-α in cancer development, common functional polymorphisms (rs1800629 and rs361525) in TNF-α have been examined extensively in many studies, but the results were inconsistent across populations, in particular the Asian population [14]–[19]. Among these studies, four studies focused on rs1800629 in an Asian population and two studies examined for rs361525, but the sample size of each study was relatively small, which limits the statistical power to determine a precise estimate. Therefore, it is necessary to increase the sample size of the Asian population and further confirm the association between these two SNPs and breast cancer susceptibility. Moreover, the SNPs in TNFRSF1A and TNFRSF1B have been widely determined in genetic association studies of various diseases [20]–[23]. However, to our knowledge, there are no published studies regarding the relationship between potentially functional SNPs in the TNFRSF1A and TNFRSF1B genes and the susceptibility to breast cancer among Asian populations. In this study, we selected seven potentially functional SNPs and determined their genotypes in a relatively large sample size. Rs1800629 (−308A/G) and rs361525 (−238A/G) in TNF-α, rs767455 (+339C/T), rs4149577 (IVS1+3420 C/T) and rs1800693 (IVS6+10 A/G) in TNFRSF1A as well as rs1061622 (+676T/G) and rs1061624 (+1663A/G) in TNFRSF1B were selected. We then examined the correlation of the seven SNPs with susceptibility to breast cancer in the Chinese Han population.

## Methods

### Ethics Statement

This study design was approved by the Medical Ethics Committee of Harbin Medical University. None of the participants were genetically related, and all of the participants provided written informed consent and 5 ml of peripheral blood.

### Subjects

This study involved 1016 sporadic breast cancer patients (mean age 50.00±8.07 years) and 806 healthy controls (mean age 48.95±7.79 years). The clinical features of the breast cancer patients, including the tumor type, lymph node metastasis and the estrogen receptor (ER), progesterone receptor (PR), human epidermal growth factor receptor 2 (C-erbB-2) and P53 statuses are summarized in Table 1. The recruitment criteria for the patients and the controls were as described previously. Briefly, all the breast cancer patients were recruited from the Third Affiliated Hospital of Harbin Medical University, Heilongjiang Province, China, and the diagnosis of breast cancer was histopathologically confirmed, the healthy controls were frequency-matched to the patients by age and were randomly selected from the routine physical examination program in the same district [24]. All the healthy subjects had no documented history of cancer or autoimmune diseases. It was ensured that the enrolled patients and controls were of the Han ethnicity. Recruitment occurred between 2007 and 2012.

**Table 1:** Clinicalpathologic features of breast cancer patients.

Feature	Case no (%)
Tumor type	
IDC	832(81.89)
Intraductal carcinoma	92(9.06)
Mucinous adenocarcinoma	22(2.17)
Invasive lobular carcinoma	22(2.17)
Others	48(4.72)
ER	
Positive	550(54.13)
Negative	299(29.43)
Unknown	167(16.44)
PR	
Positive	572(56.30)
Negative	275(27.07)
Unknown	169(16.63)
C-erbB-2	
Positive	298(29.33)
Negative	549(54.04)
Unknown	169(16.63)
P53	
Positive	231(22.74)
Negative	601(59.15)
Unknown	184(18.11)
LN involvement	
Positive	350(34.45)
Negative	532(52.36)
Unknown	134(13.19)

Abbreviations: IDC = infiltrating duct carcinoma; ER = estrogen receptor; PR = progesterone receptor; LN = lymph node; C-erbB-2 = human epidermal growth factor receptor 2.

### SNP selection and genotyping

We screened the NCBI dbSNP database (http://www.ncbi.nlm.nih.gov/snp/) for common, potentially functional SNPs with a minor allele frequency (MAF) ≥0.05 in the CHB population. SNPs located within a putative functional region of the gene (promoter, exon or 3′-UTR) were selected. Moreover, SNPs that had been previously reported to be correlated with cancer or an inflammatory disorder were also selected. Finally, a total of seven SNPs were selected, including two SNPs in TNF-α, −308 A/G (rs1800629) and −238A/G (rs361525), two SNPs in TNFRSF1B, +676T/G (rs1061622) and +1663A/G (rs1061624), as well as +339C/T (rs767455), IVS1+3420 C/T (rs4149577) and IVS6+10 A/G (rs1800693) in TNFRSF1A. The locations of the seven SNPs in gene structure are shown in Figure S1.

SNP genotyping was performed as described previously with a slight modification. Briefly, genomic DNA was isolated from blood lymphocytes using the universal genomic DNA Extraction Kit VER.3.0 (TaKaRa, Japan) following manufacturer’s procedures [24]. The DNA samples were routinely stored at −20°C. The genotyping of all seven SNPs was performed via the polymerase chain reaction-restriction fragment length polymorphism (PCR-RFLP) method, and the accuracy of the genotyping results was confirmed by the direct sequencing of random samples. The fragments containing the SNPs sites were amplified by PCR using a T-Gradient Thermoblock PCR System (Biometra, Germany). A 25 ul reaction system was selected, consisting of 0.3 µg of genomic DNA, 2.5 µl 10× PCR buffer (Mg2+ plus), 2.0 µl dNTPs mixture, 0.1 µl of each primer (Invitrogen, USA) and 2.5U TaqDNA polymerase (TaKaRa, Japan) [24]. The primers, annealing temperatures, restriction enzymes and product lengths for the PCR-RFLP are shown in Table S11. After the PCR reaction, 5 µl of product from each sample was digested with the corresponding restriction enzymes (NEB, USA), and separated in a 2% agarose gel (for rs767455, rs4149577, rs1800693, rs1061622 and rs1061624) or a 3% agarose gel (for rs1800629 and rs361525).

### Statistical analysis

The statistical analysis was performed as described previously with a slight modification. Briefly, the Hardy-Weinberg equilibrium (HWE) for the distribution of the seven SNPs was analyzed using the goodness-of-fit χ2 test by comparing the observed genotype frequencies with the expected frequencies in both the cases and controls. The genotypic associations were analyzed with three genetic models, including the codominant model, the dominant model and the recessive model [24]. The comparisons of the distributions of the alleles and genotypes were calculated using the Chi-square test or Fisher’s exact test, and the associations between the SNPs and breast cancer risk were determined by the odds ratio (OR) and the corresponding 95% confidence interval (CI) [24]. Statistical analyses were performed using the SPSS 16.0 software and the threshold for significance was set at P<0.05. We used the Haploview 4.1 program to construct haplotypes and analyze their frequencies in both cases and controls. To correct multiple testing, P-values were permutated 10,000 times using the Haploview program.

## Results

The genotype distributions of the seven polymorphisms were consistent with the Hardy-Weinberg equilibrium for both the patient and control groups (P>0.05). The distributions of the genotypes and alleles, and their associations with breast cancer are presented in Tables 2 and 3, respectively.

**Table 2:** Genotype distribution of TNF-α, TNFRSF1A and TNFRSF1B gene polymorphisms.

SNP	Genotype	Cases N (%)	Controls N (%)	OR (95% CI)	P value
TNF-α	GG	927(91.24)	716(88.83)	reference	
rs1800629	AG	89(8.76)	89(11.04)	0.764(0.560,1.042)	0.088
promoter	AA	0	1(0.12)		
	Dominant*			0.764(0.561,1.040)	0.087
rs361525	GG	928(91.34)	737(91.44)	reference	
promoter	AG	86(8.46)	68(8.44)	1.004(0.720,1.400)	0.979
	AA	2(0.20)	1(0.12)	1.588(0.144,17.551)	1.000
	Dominant			1.013(0.728,1.408)	0.939
	Recessive#			1.588(0.144,17.542)	1.000
TNFRSF1A	TT	762(75.00)	620(76.92)	reference	
rs767455	CT	236(23.23)	176(21.84)	1.091(0.874,1.362)	0.442
exon1	CC	18(1.77)	10(1.24)	1.465(0.671,3.196)	0.335
	Dominant			1.111(0.895,1.380)	0.341
	Recessive			1.436(0.659,3.128)	0.360
rs4149577	CC	370(36.42)	308(38.21)	reference	
intron1	CT	529(52.07)	381(47.27)	1.156 (0.946,1.412)	0.157
	TT	117(11.52)	117(14.52)	0.832(0.618,1.121)	0.227
	Dominant			1.080(0.892,1.307)	0.431
	Recessive			0.766(0.582,1.009)	0.057
rs1800693	AA	797(78.44)	625(77.54)	reference	
intron6	AG	203(19.98)	174(21.29)	0.915(0.728,1.149)	0.444
	GG	16(1.57)	7(0.87)	1.792(0.733,4.384)	0.195
	Dominant			0.949(0.759,1.186)	0.644
	Recessive			1.826(0.748,4.461)	0.180
TNFRSF1B	TT	687(67.62)	482(59.80)	reference	
rs1061622	GT	290(28.54)	288(35.73)	0.706(0.578,0.863)	0.000662
exon6	GG	39(3.84)	36(4.47)	0.760(0.476,1.213)	0.249
	Dominant			0.712(0.588,0.864)	0.000549
	Recessive			0.854(0.538,1.356)	0.503
rs1061624	GG	324(31.89)	322(39.95)	reference	
3′-UTR	AG	516(50.79)	365(45.29)	1.470(1.112,1.943)	0.007
	AA	176(17.32)	119(14.76)	1.405(1.145,1.724)	0.00109
	Dominant			1.421(1.171,1.724)	0.000354
	Recessive			1.210(0.939,1.559)	0.141

*The dominant model: comparing the combination of heterozygotes and minor allele homozygotes with the major allele homozygotes.

# The recessive model: comparing minor allele homozygotes with the combination of heterozygotes and major allele homozygotes.

Abbreviations: OR = odds ratio; CI = confidence interval.

**Table 3:** Allele distribution of TNF-α, TNFRSF1A and TNFRSF1B SNPs.

SNP	Allele	Case N (%)	Control N (%)	OR (95% CI)	P value
rs1800629	G	1943(95.62)	1521(94.35)	reference	
	A	89(4.38)	91(5.65)	0.766(0.567,1.033)	0.080
rs361525	G	1942(95.57)	1542(95.66)	reference	
	A	90(4.43)	70(4.34)	1.021(0.742,1.405)	0.899
rs767455	T	1760(86.61)	1416(87.84)	reference	
	C	272(13.39)	196(12.16)	1.117(0.917,1.359)	0.272
rs4149577	C	1269(62.45)	997(61.85)	reference	
	T	763(37.55)	615(38.15)	0.975(0.852,1.115)	0.710
rs1800693	A	1797(88.44)	1424(88.34)	reference	
	G	2359(11.56)	188(11.66)	0.991(0.808,1.215)	0.927
rs1061622	T	1664(81.89)	1252(77.67)	reference	
	G	368(18.11)	360(22.33)	0.769(0.654,0.905)	0.002^a^
rs1061624	G	1164(57.28)	1009(62.59)	reference	
	A	868(42.71)	603(37.41)	1.248(1.092,1.426)	0.001^b^

a P = 0.004 and ^b^P = 0.003 after correction for multiple testing.

Abbreviations: OR = odds ratio; CI = confidence interval.

### TNF-a, TNFRSF1A and TNFRSF1B gene polymorphisms and the risk of breast cancer

In TNFRSF1B, breast cancer patients who harbor the rs1061622 GT genotype had significantly reduced breast cancer risk compared with those harboring the TT genotype (P = 0.000662, OR = 0.706, 95% CI: 0.578–0.863 in Table 2). Moreover, the combined rs1061622 genotypes (GT+GG) showed a decreased risk of breast cancer under the dominant model (P = 0.000549, OR = 0.712, 95% CI: 0.588–0.864 in Table 2). In rs1061624, the AG genotype and the AA genotype were significantly associated with an increased risk in comparison with the GG genotype (P = 0.007, OR = 1.470, 95% CI: 1.112–1.943; P = 0.00109, OR = 1.405, 95% CI: 1.145–1.724, respectively in Table 2), and this association was also observed in the dominant model (AG+AA vs. GG, P = 0.000354, OR = 1.421, 95% CI: 1.171–1.724 in Table 2). In the allele analysis, we found that the rs1061622 G allele was more likely to decrease breast cancer risk compared to the T allele (P = 0.002, OR = 0.769, 95% CI: 0.654–0.905 in Table 3) and the rs1061624 A allele was more likely to increase breast cancer risk in comparison with the G allele (P = 0.001, OR = 1.248, 95% CI: 1.092–1.426 in Table 3). After the correction for multiple testing, significant associations were also observed for the rs1061622 G and the rs1061624 A alleles (P = 0.004; P = 0.003, respectively). However, we did not found any correlation between TNF-α or TNFRSF1A polymorphisms and breast cancer risk.

The distributions of the haplotypes in the breast cancer cases and healthy controls were further analyzed by Haploview software. The haplotypes that occurred with an estimated frequency of greater than 1% are shown in Table 4. The CTA (rs767455 C-rs4149577 T-rs1800693 A) haplotype in TNFRSF1A had a higher frequency in the patients than in the healthy controls (P = 0.00324). In the TNFRSF1B gene, the TA (rs1061622 T-rs1061624 A) haplotype had a higher frequency, and the GG (rs1061622 G-rs1061624 G) haplotype had a lower frequency in the patients compared to the healthy controls (TA: P = 0.000370, GG: P = 0.000251). After correcting for multiple testing, the P-value was still significant for the CTA haplotype (P = 0.0065), the TA haplotype (P = 0.0009) and the GG haplotype (P = 0.0008).

**Table 4:** Haplotypes distributions of TNF-α, TNFRSF1A and TNFRSF1B.

Gene	Haplotype	Frequency	Cases	Controls	P value
TNF-α#	GG	0.907	0.912	0.900	0.224
	AG	0.049	0.044	0.056	0.080
	GA	0.044	0.044	0.043	0.899
TNFRSF1A*	TCA	0.601	0.601	0.600	0.976
	TTA	0.256	0.249	0.265	0.290
	CTG	0.095	0.092	0.098	0.586
	CTA	0.017	0.023	0.010	0.00324^a^
	TTG	0.010	0.011	0.009	0.564
	CCA	0.010	0.012	0.008	0.349
TNFRSF1B&	TG	0.476	0.470	0.483	0.422
	TA	0.324	0.349	0.293	0.000370^b^
	GG	0.120	0.103	0.142	0.000251^c^
	GA	0.079	0.078	0.081	0.782

# The order of SNPs in TNF-α is rs1800629 and rs361525.

*The order of SNPs in TNFRSF1A is rs767455, rs4149577 and rs1800693.

& The order of SNPs in TNFRSF1A is rs1061622 and rs1061624.

a P = 0.0065, ^b^P = 0.0009 and ^c^P = 0.0008 after correction for multiple testing.

### Associations between the TNF-α, TNFRSF1A and TNFRSF1B polymorphisms and clinicopathological features of the cases

The associations between SNPs in the TNF-α, TNFRSF1A and TNFRSF1B genes and the clinicopathological features of breast cancer are shown in Tables S1–S5. However, significant associations were only observed in TNFRSF1A polymorphisms. In rs767455, the CT genotype was less frequent in the P53-positive cases in comparison with the TT genotype (P = 0.024 in Table S4). In rs4149577, compared with the CC genotype, the TT genotype occurred more frequently in both ER-and PR-positive cases (P = 0.006 in Table S1; P = 0.032 in Table S2, respectively). In rs1800693, in comparison with the AA genotype, the AG genotype had a higher frequency in the ER-positive cases and a lower frequency in P53-positive cases (P = 0.010 in Table S1; P = 0.014 in Table S4, respectively). Moreover, the patients with rs1800693 GG genotype presented a higher proportion of lymph node metastasis (P = 0.030 in Table S5). Furthermore, compared with the major allele, the frequencies of the rs4149577 T and rs1800693 G alleles were higher in ER-positive cases (P = 0.008; P = 0.011, respectively, Table S1), and the differences remained significant after correcting multiple testing (P = 0.019; P = 0.025, respectively).

The associations between the haplotypes and the clinical features of the breast cancer are shown in Table S6–S10. In the TNFRSF1A gene, the TCA (rs767455 T-rs4149577 C-rs1800693 A) haplotype was occurred less frequently in the ER-positive cases (P = 0.020 in Table S6), and remained significant after the correction for multiple testing (P = 0.045 in Table S6). The CTG (rs767455 C-rs4149577 T-rs1800693 G) haplotype was occurred more frequently in both the ER-and PR-positive cases (P = 0.008 in Table S6; P = 0.014 in Table S7, respectively), and these differences were also found after correcting the P-value (P = 0.015; P = 0.030, respectively). Moreover, the frequency of TTG (rs767455 T-rs4149577 T-rs1800693 G) haplotype was higher in those cases that were positive for lymph node involvement (P = 0.049 in Table S10), but no relationship was found after correcting the P-value.

## Discussion

Inflammatory responses play decisive roles at different stages of tumor development, including pathogenesis, invasion, and metastasis, therefore, inflammatory cytokines are critical components of tumor progression. TNF-α and its receptors, TNFRSF1A and TNFRSF1B belong to the TNF-TNFR superfamily, and the interaction of these genes regulates inflammation and increases the invasive activity and metastatic potential of tumor cells. In the current case-control study, we determined the associations between potentially functional SNPs in the TNF-α, TNFRSF1A and TNFRSF1B genes and breast cancer susceptibility.

TNF-α is a major regulator of the inflammatory response through inducing the expression of other pro-inflammatory and chemotactic cytokines and adhesion factors. TNF-α is also produced by neoplastic cells or cells in the tumor microenvironment and can act as an endogenous tumor promoter. In normal breast tissue, TNF-α regulates cell proliferation through its pro-apoptotic effects, but in breast cancer, the inhibition of the apoptotic pathway and the enhancement of the survival and proliferation effects contribute to tumor cell proliferation [9]. The SNPs within TNF-α promoter have been investigated widely in relation to breast cancer, and the most frequently studied variants are rs1800629 and rs361525, which have been observed to affect TNF-α transcriptional activity [25], [26]. Two meta-analysis studies revealed that rs1800629 was significantly associated with breast cancer risk in Caucasian individuals when the cohort was stratified by ethnicity, and no association between rs361525 and breast cancer risk was found in any population [27], [28]. In our present study, we first investigated these associations in a Chinese Han population consisting of 1016 breast cancer patients and 806 healthy controls, but no significant association was found, which is in consistent with the results of the meta-analysis of the Asian population [26]. There is a difference between the sample size in our study and the previous study with a Caucasian population. However, according to the power calculations, the sample size of our study had adequate statistical power. Therefore, these conflicting results between Caucasian individuals and Asian individuals may be partly explained by the genetic heterogeneity among the different ethnic groups.

As one of the receptors for TNF-α, TNFRSF1A can activate NF-KappaB and regulate inflammation. Rs767455 is a synonymous SNP and is located in exon1 of TNFRSF1A. Synonymous SNPs may generate ectopic mRNA splicing, alter the structure of the mRNA and affect protein folding [29]. Accordingly, Madeleine et al suggested that this SNP was associated with breast cancer risk in Caucasian women [30]. However, our data showed no association between this SNP and the susceptibility to breast cancer in a Chinese population. Thus, the association between this SNP and breast cancer susceptibility requires confirmation in other populations. Rs4149577 and rs1800693 are located in the intron1 and intron6, respectively, and these two SNPs have been investigated widely regarding their associations with autoimmune diseases. The results of many studies and two recently published meta-analyses of studies indicate that rs1800693 is associated with an increased risk of Multiple Sclerosis (MS) [20], [22]. A variety of studies also provided evidence that rs4149577 can affect ankylosing spondylitis (AS) risk [21], [23]. In our study, however, neither of these two SNPs was associated with breast cancer risk, which indicates that these two SNPs exert their functions only in autoimmune diseases and not in cancers.

As another receptor of TNF-α, TNFRSF1B could enhance the role of TNFRSF1A in TNF-mediated toxicity [31]. TNFRSF1B expressed higher in infiltrating breast tumors, which suggest that increased TNFRSF1B expression would be a factor for a poor prognosis in breast cancer patients [9]. Rs1061622 is located in the exon6 of TNFRSF1B, and the change of T to G causes a functional amino acid substitution at codon196 from methionine (Met) to arginine (Arg). This variant is supposed to produce a soluble form of TNFRSF1B, impair NF-kappaB signaling and affect TNF-α-induced apoptosis [32]. Moreover, in some drug sensitivity studies, rs1061622 was found to be associated with the prognosis of non-small cell lung cancer patients treated with chemoradiotherapy [33] and with a beneficial response to infliximab (IFX) in Crohn’s disease [34]. Consistent with the above results, our study also found an association between rs1061622 and breast cancer risk. Thus, we speculate that this SNP may alter the protein coding sequences and further affect the biological function of the protein. In our study, the rs1061624 AG and GG genotypes were observed to be associated with an increased risk of breast cancer. The rs1061624 is located in the 3′-UTR of the TNFRSF1B gene. SNPs in the 3′UTR sites targeted by miRNAs can create or destroy the microRNA-binding sites, regulate the expression of target gene, and further affect process of carcinogenesis [35]. Rs1061624 was speculated to be located in some miRNA-binding sites using the MicroSNiPer software (http://cbdb.nimh.nih.gov/microsniper/index.php). In line with our research, this SNP was also found to be associated with the susceptibility to inflammatory diseases, such as tuberculosis and ulcerative colitis [36], [37]. These results cause us to speculate that this SNP may affect binding strength of miRNAs, cause a subsequent dysregulation of the TNFRSF1B gene stability and ultimately result in an altered risk for breast cancer.

In haplotype analysis, we found that the CTA haplotype in TNFRSF1A and TA haplotype in TNFRSF1B were more frequent in the breast cancer cases, and the, GG haplotype in TNFRSF1B had lower frequency in the breast cancer patients. Therefore, the CTA haplotype and TA haplotype may be important in increasing breast cancer risk, whereas the GG haplotype may have protective effect against breast cancer.

The clinicopathological features can predict breast cancer prognosis and treatment outcomes. ER and PR-positive patients have a considerably better prognosis than ER and PR-negative patients when the patients are treated with endocrine therapy. These two statuses have been more commonly used as predictive markers for endocrine therapy [38], [39]. Patients with a mutation in P53 and lymph node metastasis are insensitive to endocrine therapy and had higher recurrence rates and poor prognosis [40], [41]. Our data showed that rs4149577 and rs1800693 were related to ER status, and rs4149577 was related to PR status. Moreover, rs767455 and rs1800693 were observed to be related to P53 status, and there was also a correlation between rs1800693 and lymph node involvement. Therefore, our data suggest that rs4149577, rs767455 and rs1800693 may be important in predicting the prognosis of breast cancer patients. In the haplotype analysis for TNFRSF1A, the TCA haplotype and the TTG haplotype were observed to be related to the ER status and lymph node involvement, and the CTG haplotype had an association with both the ER and PR statuses. Therefore, these three haplotypes may be meaningful in the pathology of breast cancer and provide valuable prognostic information for breast cancer patients.

## Conclusion

Taken together, the current study showed that the rs1061622 and rs1061624 in TNFRSF1B as well as some haplotypes might affect breast cancer susceptibility. The SNPs in TNFRSF1A were associated with the clinicopathological features of breast cancer. These findings will be important in further understanding the role of TNF-α and its receptors in breast cancer development.

## Supporting Information

Figure S1 **Gene structure of TNF-α, TNFRSF1A and TNFRSF1B.** (A) Gene structure of TNF-α. Rs1800629 and rs361525 are both located in the promoter of TNF-α. (B) Gene structure of TNFRSF1A. Rs767455 is located in the exon1, rs4149577 is located in the intron1 and rs1800693 is located in the intron6. (C) Gene structure of TNFRSF1B. Rs1061622 is located in the exon6 and rs1061624 is located in the 3′-UTR.
(TIF)

Table S1 **Associations between TNF-α, TNFRSF1A and TNFRSF1B SNPs and ER status.**
(DOC)

Table S2 **Associations between TNF-α, TNFRSF1A and TNFRSF1B SNPs and PR status.**
(DOC)

Table S3 **Associations between TNF-α, TNFRSF1A and TNFRSF1B SNPs and C-erbB-2 status.**
(DOC)

Table S4 **Associations between TNF-α, TNFRSF1A and TNFRSF1B SNPs and P53 status.**
(DOC)

Table S5 **Associations between TNF-α, TNFRSF1A and TNFRSF1B SNPs and LN involvement.**
(DOC)

Table S6 **Association between TNF-α, TNFRSF1A and TNFRSF1B haplotypes and ER status.**
(DOC)

Table S7 **Associations between TNF-α, TNFRSF1A and TNFRSF1B haplotypes and PR status.**
(DOC)

Table S8 **Associations between TNF-α, TNFRSF1A and TNFRSF1B haplotypes and C-erbB-2 status.**
(DOC)

Table S9 **Associations between TNF-α, TNFRSF1A and TNFRSF1B haplotypes and P53 status.**
(DOC)

Table S10 **Associations between TNF-α, TNFRSF1A and TNFRSF1B haplotypes and LN involvement.**
(DOC)

Table S11 **Genotyping information for TNF-α, TNFRSF1A and TNFRSF1B SNPs.**
(DOC)
