# Supplementary figures and images for: Association of TNF-α, TNFRSF1A and TNFRSF1B Gene Polymorphisms with the Risk of Sporadic Breast Cancer in Northeast Chinese Han Women
Source: PLoS One. 2014 Jul 10;9(7):e101138. doi: 10.1371/journal.pone.0101138 (PMC4091942; doi:10.1371/journal.pone.0101138)

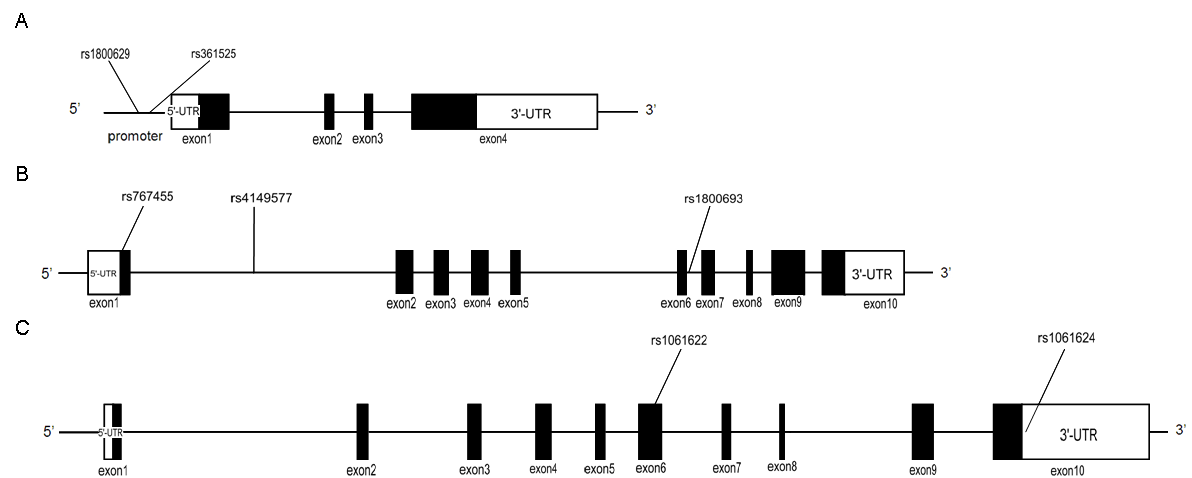

Supplement: Figure S1 — Gene structure of TNF-α, TNFRSF1A and TNFRSF1B. (A) Gene structure of TNF-α. Rs1800629 and rs361525 are both located in the promoter of TNF-α. (B) Gene structure of TNFRSF1A. Rs767455 is located in the exon1, rs4149577 is located in the intron1 and rs1800693 is located in the intron6. (C) Gene structure of TNFRSF1B. Rs1061622 is located in the exon6 and rs1061624 is located in the 3′-UTR. (TIF) [file pone.0101138.s001.tif]
