# Supplementary material for: Association of TNF-α, TNFRSF1A and TNFRSF1B Gene Polymorphisms with the Risk of Sporadic Breast Cancer in Northeast Chinese Han Women
Source: PLoS One. 2014 Jul 10;9(7):e101138. doi: 10.1371/journal.pone.0101138 (PMC4091942; doi:10.1371/journal.pone.0101138)
Supplement: Table S1 — Associations between TNF-α, TNFRSF1A and TNFRSF1B SNPs and ER status. (DOC) [file pone.0101138.s002.doc]

Table S1. Associations between TNF-α, TNFRSF1A and TNFRSF1B SNPs and ER status

| SNP | Genotype and allele | Positive  N (%) | Negative  N (%) | OR (95% CI) | P value |
| --- | --- | --- | --- | --- | --- |
| TNF-α  rs1800629 | GG | 497(90.36) | 276 (92.31) | reference |  |
|  | AG | 53(9.64) | 23(7.69) | 1.280(0.768,2.133) | 0.343 |
|  | AA | 0 | 0 |  |  |
|  | G | 1047(95.18) | 575(95.65) | reference |  |
|  | A | 53(4.82) | 23(3.85) | 1.266(0.768,2.086) | 0.355 |
| rs361525 | GG | 503(91.45) | 278(92.98) | reference |  |
|  | AG | 47(8.55) | 20(6.69) | 1.299(0.754,2.236) | 0.345 |
|  | AA | 0 | 1(0.33) |  |  |
|  | G | 1053(95.73) | 576(96.32) | reference |  |
|  | A | 47(4.23) | 22(3.68) | 1.169(0.697,1.959) | 0.554 |
| TNFRSF1A  rs767455 | TT | 400(72.73) | 236(78.93) | reference |  |
|  | CT | 141(25.64) | 59(19.73) | 1.410(1.000,1.989) | 0.050 |
|  | CC | 9(1.64) | 4(1.34) | 1.328(0.404,4.363) | 0.776 |
|  | T | 941(85.55) | 531(88.80) | reference |  |
|  | C | 159 (14.45) | 67(11.20) | 1.339(0.987,1.816) | 0.060 |
| rs4149577 | CC | 182(30.09) | 122(40.8) | reference |  |
|  | CT | 293(53.27) | 152(50.84) | 1.292(0.956,1.747) | 0.096 |
|  | TT | 75(13.64) | 25(8.36) | 2.011(1.211,3.340) | 0.006 |
|  | C | 657(59.73) | 396(66.22) | reference |  |
|  | T | 443(40.27) | 202(33.78) | 1.322(1.074,1.627) | 0.008^a^ |
| rs1800693 | AA | 413(75.09) | 248(82.94) | reference |  |
|  | AG | 129(23.45) | 48(16.05) | 1.614(1.118,2.329) | 0.010 |
|  | GG | 8(1.45) | 3(1.00) | 1.601(0.421,6.092) | 0.755 |
|  | A | 955(86.82) | 544(90.97) | reference |  |
|  | G | 145(13.18) | 54(9.03) | 1.530(1.100,2.127) | 0.011^b^ |
| TNFRSF1B  rs1061622 | TT | 373(67.82) | 197(65.89) | reference |  |
|  | GT | 155(28.18)) | 89(29.77) | 0.920(0.673,1.257) | 0.600 |
|  | GG | 22(4.00) | 13(4.35) | 0.894(0.441,1.813) | 0.756 |
|  | T | 901(81.91) | 483(80.77) | reference |  |
|  | G | 199(18.09) | 115(19.23) | 0.928(0.719,1.197) | 0.563 |
| rs1061624 | GG | 184(33.45) | 87(29.10) | reference |  |
|  | AG | 267(48.55) | 167(55.85) | 0.756(0.549,1.041) | 0.087 |
|  | AA | 99(18.00) | 45(15.05) | 1.040(0.673,1.608) | 0.859 |
|  | G | 635(57.73) | 341(57.02) | reference |  |
|  | A | 465(42.27) | 257(42.98) | 0.972(0.794,1.188) | 0.779 |

^a^P=0.019 and ^b^P=0.025 after correction for multiple testing

Abbreviations: OR=odds ratio; CI=confidence interval.
