# Supplementary material for: Association of TNF-α, TNFRSF1A and TNFRSF1B Gene Polymorphisms with the Risk of Sporadic Breast Cancer in Northeast Chinese Han Women
Source: PLoS One. 2014 Jul 10;9(7):e101138. doi: 10.1371/journal.pone.0101138 (PMC4091942; doi:10.1371/journal.pone.0101138)
Supplement: Table S2 — Associations between TNF-α, TNFRSF1A and TNFRSF1B SNPs and PR status. (DOC) [file pone.0101138.s003.doc]

Table S2. Associations between TNF-α, TNFRSF1A and TNFRSF1B SNPs and PR status

| SNP | Genotype and allele | Positive  N (%) | Negative  N (%) | OR (95% CI)^$^ | P value |
| --- | --- | --- | --- | --- | --- |
| TNF-α  rs1800629 | GG | 519(90.73) | 252(91.64) | reference |  |
|  | AG | 53(9.27) | 23(8.36) | 1.119(0.671,1.867) | 0.667 |
|  | AA | 0 | 0 |  |  |
|  | G | 1091(95.37) | 527(95.82) | reference |  |
|  | A | 53(4.63) | 23(4.18) | 1.113(0.675,1.836) | 0.675 |
| rs361525 | GG | 529(92.48) | 250 (90.91) | reference |  |
|  | AG | 43(7.52) | 24(8.73) | 0.847(0.503,1.426) | 0.531 |
|  | AA | 0 | 1(0.36) |  |  |
|  | G | 1101(96.24) | 524(95.27) | reference |  |
|  | A | 43(3.76) | 26(4.72) | 0.787(0.478,1.295) | 0.345 |
| TNFRSF1A  rs767455 | TT | 418(73.08) | 216(78.55) | reference |  |
|  | CT | 143(25.00) | 57(20.73) | 1.296(0.915,1.836) | 0.143 |
|  | CC | 11(1.92) | 2(0.73) | 2.842(0.624,12.937) | 0.237 |
|  | T | 979(85.58) | 489(88.91) | reference |  |
|  | C | 165(14.42) | 61(11.09) | 1.351(0.988,1.848) | 0.0589 |
| rs4149577 | CC | 201(35.14) | 102(37.09) | reference |  |
|  | CT | 294(51.4) | 151(54.91) | 0.988(0.726,1.345) | 0.939 |
|  | TT | 77(13.46) | 22(8.00) | 1.776(1.045,3.019) | 0.032 |
|  | C | 696(60.84) | 355(64.55) | reference |  |
|  | T | 448(39.16) | 195(35.45) | 1.172(0.949,1.447) | 0.141 |
| rs1800693 | AA | 437(76.4) | 223 (81.09) | reference |  |
|  | AG | 125(21.85) | 51(18.55) | 1.251(0.870,1.799) | 0.227 |
|  | GG | 10(1.75) | 1(0.36) | 5.103(0.649,40.116) | 0.111 |
|  | A | 999(87.33) | 497(90.36) | reference |  |
|  | G | 145(12.67) | 53(9.64) | 1.361(0.976,1.899) | 0.0684 |
| TNFRSF1B  rs1061622 | TT | 388(76.39) | 182(66.18) | reference |  |
|  | GT | 163(28.5) | 79(28.73) | 0.968(0.702,1.334) | 0.842 |
|  | GG | 21(3.67) | 14(5.09) | 0.704(0.350,1.415) | 0.322 |
|  | T | 939(82.08) | 443(80.55) | reference |  |
|  | G | 205(17.92) | 107(19.45) | 0.904(0.697,1.172) | 0.445 |
| rs1061624 | GG | 190(33.22) | 81(29.45) | reference |  |
|  | AG | 273(47.73) | 159(57.82) | 0.732(0.529,1.013) | 0.060 |
|  | AA | 109(19.06) | 35(12.73) | 1.328(0.837,2.106) | 0.228 |
|  | G | 653(57.08) | 321(58.36) | reference |  |
|  | A | 491(42.92) | 229(41.64) | 1.054(0.858,1.295) | 0.617 |

Abbreviations: OR=odds ratio; CI=confidence interval.
