# Supplementary material for: Association of TNF-α, TNFRSF1A and TNFRSF1B Gene Polymorphisms with the Risk of Sporadic Breast Cancer in Northeast Chinese Han Women
Source: PLoS One. 2014 Jul 10;9(7):e101138. doi: 10.1371/journal.pone.0101138 (PMC4091942; doi:10.1371/journal.pone.0101138)
Supplement: Table S3 — Associations between TNF-α, TNFRSF1A and TNFRSF1B SNPs and C-erbB-2 status. (DOC) [file pone.0101138.s004.doc]

Table S3. Associations between TNF-α, TNFRSF1A and TNFRSF1B SNPs and C-erbB-2 status

| SNP | Genotype | Positive  N (%) | Negative  N (%) | OR (95% CI) | P value |
| --- | --- | --- | --- | --- | --- |
| TNF-α  rs1800629 | GG | 273(42.24) | 498(90.71) | reference |  |
|  | AG | 25(45.21) | 51(9.29) | 0.893(0.542,1.474) | 0.659 |
|  | AA | 0 | 0 |  |  |
|  | G | 571(95.47) | 1047(95.36) | reference |  |
|  | A | 25(36.89) | 51(4.65) | 0.899(0.551,1.466) | 0.669 |
| rs361525 | GG | 276(92.62) | 503(91.62) | reference |  |
|  | AG | 22(7.38) | 45(8.2) | 0.891 (0.524,1.515) | 0.670 |
|  | AA | 0 | 1(0.18) |  |  |
|  | G | 574(96.31) | 1051(95.72) | reference |  |
|  | A | 22(3.69) | 47(4.28) | 0.862(0.514,1.444) | 0.572 |
| TNFRSF1A  rs767455 | TT | 225(75.5) | 409(74.5) | reference |  |
|  | CT | 71(23.83) | 129(23.5) | 1.000(0.718,1.395) | 0.998 |
|  | CC | 2(0.67) | 11(2) | 0.331(0.073,1.504) | 0.155 |
|  | T | 521 (87.42) | 947(86.25) | reference |  |
|  | C | 75(12.58) | 151(13.75) | 0.903(0.671,1.205) | 0.499 |
| rs4149577 | CC | 100(33.56) | 203(36.98) | reference |  |
|  | CT | 163(54.7) | 282(51.37) | 1.173(0.863,1.596) | 0.308 |
|  | TT | 35(11.74) | 64(11.66) | 1.110(0.689,1.788) | 0.667 |
|  | C | 363(60.91) | 688(62.66) | reference |  |
|  | T | 233(39.09) | 410(37.34) | 1.077(0.877,1.322) | 0.478 |
| rs1800693 | AA | 238(79.87) | 422 (76.87) | reference |  |
|  | AG | 58(19.46) | 118(21.49) | 0.872(0.613,1.239) | 0.444 |
|  | GG | 2(0.67) | 9(1.64) | 0.394(0.084,1.839) | 0.344 |
|  | A | 534(89.60) | 962(87.61) | reference |  |
|  | G | 62(10.40) | 136(12.39) | 0.821(0.597,1.129) | 0.225 |
| TNFRSF1B  rs1061622 | TT | 200(67.11) | 370(67.4) | reference |  |
|  | GT | 88(29.53) | 154(28.05) | 1.057(0.773,1.446) | 0.728 |
|  | GG | 10(3.36) | 25(4.55) | 0.740(0.348,1.572) | 0.432 |
|  | T | 488(81.88) | 894(81.42) | reference |  |
|  | G | 108(18.12) | 204(18.58) | 0.970(0.749,1.255) | 0.816 |
| rs1061624 | GG | 105(35.23) | 166(30.24) | reference |  |
|  | AG | 142(47.65) | 290(52.82) | 0.774(0.564,1.062) | 0.112 |
|  | AA | 51(17.11) | 93(16.94) | 0.867 (0.570,1.319) | 0.505 |
|  | G | 352(59.06) | 622(56.65) | reference |  |
|  | A | 244(40.94) | 476(43.35) | 0.906(0.740,1.109) | 0.338 |

Abbreviations: OR=odds ratio; CI=confidence interval.
