# Supplementary material for: Association of TNF-α, TNFRSF1A and TNFRSF1B Gene Polymorphisms with the Risk of Sporadic Breast Cancer in Northeast Chinese Han Women
Source: PLoS One. 2014 Jul 10;9(7):e101138. doi: 10.1371/journal.pone.0101138 (PMC4091942; doi:10.1371/journal.pone.0101138)
Supplement: Table S4 — Associations between TNF-α, TNFRSF1A and TNFRSF1B SNPs and P53 status. (DOC) [file pone.0101138.s005.doc]

Table S4. Associations between TNF-α, TNFRSF1A and TNFRSF1B SNPs and P53 status

| SNP | Genotype | Positive  N (%) | Negative  N (%) | OR (95% CI) | P value |
| --- | --- | --- | --- | --- | --- |
| TNF-α  rs1800629 | GG | 212(91.77) | 546(90.85) | reference |  |
|  | AG | 19(8.23) | 55(9.14) | 0.890(0.516,1.535) | 0674 |
|  | AA | 0 | 0 |  |  |
|  | G | 443(95.89) | 1147(95.42) | reference |  |
|  | A | 19(4.11) | 55(4.58) | 0.894(0.525,1.524) | 0.681 |
| rs361525 | GG | 213(92.21) | 553(92.01) | reference |  |
|  | AG | 18(7.79) | 47(7.82) | 0.994(0.565,1.751) | 0.984 |
|  | AA | 0 | 1(0.17) |  |  |
|  | G | 444(96.10) | 1153(95.92) | reference |  |
|  | A | 18(3.90) | 49(4.08) | 0.954(0.550,1.655) | 0.867 |
| TNFRSF1A  rs767455 | TT | 185(80.09) | 438(72.88) | reference |  |
|  | CT | 42(18.18) | 154(25.62) | 0.646(0.441,0.946) | 0.024 |
|  | CC | 4(1.73) | 9(1.5) | 1.052(0.320,3.460) | 0.933 |
|  | T | 412(89.18) | 1030(85.69) | reference |  |
|  | C | 50(10.82) | 172(14.31) | 0.727(0.520,1.016) | 0061 |
| rs4149577 | CC | 83(35.93) | 214(35.61) | reference |  |
|  | CT | 119(51.52) | 320(53.24) | 0.959(0.690,1.334) | 0.802 |
|  | TT | 29(12.55) | 67(11.15) | 1.116(0.674,1.847) | 0.669 |
|  | C | 285(61.69) | 748(65.22) | reference |  |
|  | T | 177(38.31) | 454(37.77) | 1.023(0.820,1.276) | 0.839 |
| rs1800693 | AA | 192(83.12) | 456(75.87) | reference |  |
|  | AG | 35(15.15) | 138(22.96) | 0.602(0.401,0.905) | 0.014 |
|  | GG | 4(1.73) | 7(1.16) | 1.357(0.393,4.690) | 0.740 |
|  | A | 419(90.69) | 1050(87.35) | reference |  |
|  | G | 43(9.31) | 152(12.65) | 0.709(0.496,1.013) | 0.058 |
| TNFRSF1B  rs1061622 | TT | 155(67.1) | 406(67.55) | reference |  |
|  | GT | 70(30.3)) | 166(27.62) | 1.105(0.790,1.544) | 0.561 |
|  | GG | 6(2.6) | 29(4.83) | 0.542(0.221,1.331) | 0.175 |
|  | T | 380(82.25) | 978(81.36) | reference |  |
|  | G | 82(17.75) | 224(18.64) | 0.942(0.713,1.246) | 0.676 |
| rs1061624 | GG | 78(33.77) | 190(31.61) | reference |  |
|  | AG | 117(50.65) | 306(50.92) | 0.931(0.664,1.307) | 0.681 |
|  | AA | 36(15.58) | 105(17.47) | 0.835(0.527,1.325) | 0.444 |
|  | G | 273(59.09) | 686(57.07) | reference |  |
|  | A | 189(40.91) | 516(42.93) | 0.920(0.740,1.144) | 0.455 |

Abbreviations: OR=odds ratio; CI=confidence interval.
