# Supplementary material for: Association of TNF-α, TNFRSF1A and TNFRSF1B Gene Polymorphisms with the Risk of Sporadic Breast Cancer in Northeast Chinese Han Women
Source: PLoS One. 2014 Jul 10;9(7):e101138. doi: 10.1371/journal.pone.0101138 (PMC4091942; doi:10.1371/journal.pone.0101138)
Supplement: Table S5 — Associations between TNF-α, TNFRSF1A and TNFRSF1B SNPs and LN involvement. (DOC) [file pone.0101138.s006.doc]

Table.S5. Associations between TNF-α, TNFRSF1A and TNFRSF1B SNPs and LN involvement

| SNP | Genotype | Positive  N (%) | Negative  N (%) | OR (95% CI) | P value |
| --- | --- | --- | --- | --- | --- |
| TNF-α  rs1800629 | GG | 323(92.29) | 478(89.85) | reference |  |
|  | AG | 27(7.71) | 54(10.15) | 0.740(0.456,1.199) | 0.220 |
|  | AA | 0 | 0 |  |  |
|  | G | 673(96.14) | 1010(94.92) | reference |  |
|  | A | 27(3.86) | 54(5.08) | 0.750(0.468,1203) | 0.232 |
| rs361525 | GG | 314(89.71) | 495(93.05) | reference |  |
|  | AG | 36(10.29) | 36(6.77) | 1.576(0.972,2.556) | 0.063 |
|  | AA | 0 | 1(0.19) |  |  |
|  | G | 664(94.86) | 1026(96.43) | reference |  |
|  | A | 36(5.14) | 38(3.57) | 1.464(0.918,2.333) | 0.107 |
| TNFRSF1A  rs767455 | TT | 269(76.86) | 397(74.62) | reference |  |
|  | CT | 72(20.57) | 130(24.44) | 0.817(0.589,1.133) | 0.226 |
|  | CC | 9(2.57) | 5(0.94) | 2.657(0.881,8.014) | 0.072 |
|  | T | 610(87.14) | 924(86.84) | reference |  |
|  | C | 90(12.86) | 140(13.16) | 0.993(0.748,1.318) | 0.960 |
| rs4149577 | CC | 137(39.14) | 185(34.77) | reference |  |
|  | CT | 169(48.29) | 286(53.76) | 0.798(0.596,1.068) | 0.129 |
|  | TT | 44(12.57) | 61(11.47) | 0.974(0.623,1.522) | 0.908 |
|  | C | 443(63.29) | 656(61.65) | reference |  |
|  | T | 257(36.71) | 408(38.35) | 0.933(0766,1.136) | 0.489 |
| rs1800693 | AA | 273(78) | 418(78.57) | reference |  |
|  | AG | 68(19.43) | 110(20.68) | 0.947(0.675,1.328) | 0.750 |
|  | GG | 9(2.57) | 4(0.75) | 3.445(1.051,12.297) | 0.030 |
|  | A | 614(87.71) | 946(88.91) | reference |  |
|  | G | 86(12.29) | 118(11.09) | 1.123(0.835,1.510) | 0.442 |
| TNFRSF1B  rs1061622 | TT | 237(67.71) | 363(68.23) | reference |  |
|  | GT | 102(29.14) | 148(27.82) | 1.056(0.781,1.426) | 0.724 |
|  | GG | 11(3.14) | 21(3.95) | 0.802(0.380,1.694) | 0.563 |
|  | T | 576(82.29) | 874(82.14) | reference |  |
|  | G | 124(17.71) | 190(17.86) | 0.990(0.772,1.271) | 0.939 |
| rs1061624 | GG | 108(30.86) | 169(31.77) | reference |  |
|  | AG | 179(51.14) | 276(51.88) | 1.015(0.747,1.378) | 0.925 |
|  | AA | 63(18) | 87(16.35) | 1.133(0.756,1.698) | 0.544 |
|  | G | 395(56.43) | 614(57.71) | reference |  |
|  | A | 305(43.57) | 450(42.29) | 1.054(0.869,1.277) | 0.596 |

Abbreviations: OR=odds ratio; CI=confidence interval.
