# Supplementary material for: Association of TNF-α, TNFRSF1A and TNFRSF1B Gene Polymorphisms with the Risk of Sporadic Breast Cancer in Northeast Chinese Han Women
Source: PLoS One. 2014 Jul 10;9(7):e101138. doi: 10.1371/journal.pone.0101138 (PMC4091942; doi:10.1371/journal.pone.0101138)
Supplement: Table S6 — Association between TNF-α, TNFRSF1A and TNFRSF1B haplotypes and ER status. (DOC) [file pone.0101138.s007.doc]

Table S6. Association between TNF-α, TNFRSF1A and TNFRSF1B haplotypes and ER status

| Gene | Haplotype | Frequency | Positive | Negative | P value |
| --- | --- | --- | --- | --- | --- |
| TNF-α^#^ | GG | 0.915 | 0.909 | 0.925 | 0.270 |
|  | AG | 0.045 | 0.048 | 0.038 | 0.355 |
|  | GA | 0.041 | 0.043 | 0.037 | 0.554 |
| TNFRSF1A^*^ | TCA | 0.598 | 0.577 | 0.635 | 0.020^a^ |
|  | TTA | 0.255 | 0.263 | 0.241 | 0.311 |
|  | CTG | 0.096 | 0.110 | 0.070 | 0.008^b^ |
|  | CTA | 0.019 | 0.018 | 0.021 | 0.692 |
|  | CCA | 0.010 | 0.009 | 0.013 | 0.528 |
| TNFRSF1B^&^ | TG | 0.468 | 0.469 | 0.466 | 0.919 |
|  | TA | 0.347 | 0.350 | 0.342 | 0.715 |
|  | GG | 0.107 | 0.109 | 0.104 | 0.776 |
|  | GA | 0.078 | 0.072 | 0.088 | 0.244 |

^#^ The order of SNPs in TNF-α is rs1800629 and rs361525.

^*^The order of SNPs in TNFRSF1A is rs767455, rs4149577 and rs1800693.

^&^The order of SNPs in TNFRSF1A is rs1061622 and rs1061624.

^a^P=0.045, ^b^P=0.015 after correction for multiple testing
