# Supplementary material for: Association of TNF-α, TNFRSF1A and TNFRSF1B Gene Polymorphisms with the Risk of Sporadic Breast Cancer in Northeast Chinese Han Women
Source: PLoS One. 2014 Jul 10;9(7):e101138. doi: 10.1371/journal.pone.0101138 (PMC4091942; doi:10.1371/journal.pone.0101138)
Supplement: Table S7 — Associations between TNF-α, TNFRSF1A and TNFRSF1B haplotypes and PR status. (DOC) [file pone.0101138.s008.doc]

Table S7 Association between TNF-α, TNFRSF1A and TNFRSF1B haplotypes and PR status

| Gene | Haplotype | Frequency | Positive | Negative | P value |
| --- | --- | --- | --- | --- | --- |
| TNF-α^#^ | GG | 0.914 | 0.916 | 0.911 | 0.732 |
|  | AG | 0.045 | 0.046 | 0.042 | 0.675 |
|  | GA | 0.041 | 0.038 | 0.047 | 0.345 |
| TNFRSF1A^*^ | TCA | 0.598 | 0.587 | 0.619 | 0.212 |
|  | TTA | 0.256 | 0.257 | 0.251 | 0.788 |
|  | CTG | 0.096 | 0.108 | 0.071 | 0.014^a^ |
|  | CTA | 0.019 | 0.019 | 0.020 | 0.950 |
|  | CCA | 0.010 | 0.009 | 0.013 | 0.423 |
| TNFRSF1B^&^ | TG | 0.468 | 0.462 | 0.481 | 0.919 |
|  | TA | 0.348 | 0.359 | 0.324 | 0.715 |
|  | GG | 0.107 | 0.109 | 0.102 | 0.776 |
|  | GA | 0.077 | 0.070 | 0.092 | 0.244 |

^#^ The order of SNPs in TNF-α is rs1800629 and rs361525.

^*^The order of SNPs in TNFRSF1A is rs767455, rs4149577 and rs1800693.

^&^The order of SNPs in TNFRSF1A is rs1061622 and rs1061624.

^a^P=0.030 after correction for multiple testing
