# Supplementary material for: Association of TNF-α, TNFRSF1A and TNFRSF1B Gene Polymorphisms with the Risk of Sporadic Breast Cancer in Northeast Chinese Han Women
Source: PLoS One. 2014 Jul 10;9(7):e101138. doi: 10.1371/journal.pone.0101138 (PMC4091942; doi:10.1371/journal.pone.0101138)
Supplement: Table S8 — Associations between TNF-α, TNFRSF1A and TNFRSF1B haplotypes and C-erbB-2 status. (DOC) [file pone.0101138.s009.doc]

Table S8. Association between TNF-α, TNFRSF1A and TNFRSF1B haplotypes and C-erbB-2 status

| Gene | Haplotype | Frequency | Positive | Negative | P value |
| --- | --- | --- | --- | --- | --- |
| TNF-α^#^ | GG | 0.914 | 0.921 | 0.911 | 0.465 |
|  | AG | 0.045 | 0.042 | 0.046 | 0.669 |
|  | GA | 0.041 | 0.037 | 0.043 | 0.558 |
| TNFRSF1A^*^ | TCA | 0.598 | 0.590 | 0.602 | 0.643 |
|  | TTA | 0.256 | 0.275 | 0.245 | 0.171 |
|  | CTG | 0.096 | 0.091 | 0.099 | 0.637 |
|  | CTA | 0.019 | 0.018 | 0.020 | 0.799 |
|  | CCA | 0.010 | 0.012 | 0.009 | 0.587 |
| TNFRSF1B^&^ | TG | 0.468 | 0.474 | 0.465 | 0.710 |
|  | TA | 0.348 | 0.344 | 0.349 | 0.841 |
|  | GG | 0.107 | 0.116 | 0.102 | 0.350 |
|  | GA | 0.077 | 0.065 | 0.084 | 0.157 |

^#^ The order of SNPs in TNF-α is rs1800629 and rs361525.

^*^The order of SNPs in TNFRSF1A is rs767455, rs4149577 and rs1800693.

^&^The order of SNPs in TNFRSF1A is rs1061622 and rs1061624.
