# Supplementary material for: Association of TNF-α, TNFRSF1A and TNFRSF1B Gene Polymorphisms with the Risk of Sporadic Breast Cancer in Northeast Chinese Han Women
Source: PLoS One. 2014 Jul 10;9(7):e101138. doi: 10.1371/journal.pone.0101138 (PMC4091942; doi:10.1371/journal.pone.0101138)
Supplement: Table S9 — Associations between TNF-α, TNFRSF1A and TNFRSF1B haplotypes and P53 status. (DOC) [file pone.0101138.s010.doc]

Table S9. Association between TNF-α, TNFRSF1A and TNFRSF1B haplotypes and P53 status

| Gene | Haplotype | Frequency | Positive | Negative | P value |
| --- | --- | --- | --- | --- | --- |
| TNF-α^#^ | GG | 0.915 | 0.920 | 0.913 | 0.673 |
|  | AG | 0.044 | 0.041 | 0.046 | 0.682 |
|  | GA | 0.040 | 0.039 | 0.041 | 0.867 |
| TNFRSF1A^*^ | TCA | 0.598 | 0.599 | 0.597 | 0.967 |
|  | TTA | 0.255 | 0.280 | 0.246 | 0.151 |
|  | CTG | 0.096 | 0.078 | 0.103 | 0.129 |
|  | CTA | 0.019 | 0.019 | 0.019 | 0.956 |
|  | CCA | 0.011 | 0.009 | 0.011 | 0.778 |
| TNFRSF1B^&^ | TG | 0.471 | 0.489 | 0.464 | 0.345 |
|  | TA | 0.345 | 0.333 | 0.350 | 0.515 |
|  | GG | 0.106 | 0.101 | 0.107 | 0.739 |
|  | GA | 0.078 | 0.049 | 0.076 | 0.824 |

^#^ The order of SNPs in TNF-α is rs1800629 and rs361525.

^*^The order of SNPs in TNFRSF1A is rs767455, rs4149577 and rs1800693.

^&^The order of SNPs in TNFRSF1A is rs1061622 and rs1061624.
