# Supplementary material for: Association of TNF-α, TNFRSF1A and TNFRSF1B Gene Polymorphisms with the Risk of Sporadic Breast Cancer in Northeast Chinese Han Women
Source: PLoS One. 2014 Jul 10;9(7):e101138. doi: 10.1371/journal.pone.0101138 (PMC4091942; doi:10.1371/journal.pone.0101138)
Supplement: Table S10 — Associations between TNF-α, TNFRSF1A and TNFRSF1B haplotypes and LN involvement. (DOC) [file pone.0101138.s011.doc]

Table S10. Association between TNF-α,TNFRSF1A and TNFRSF1B haplotypes and LN involvement

| Gene | Haplotype | Frequency | Positive | Negative | P value |
| --- | --- | --- | --- | --- | --- |
| TNF-α^#^ | GG | 0.912 | 0.910 | 0.914 | 0.798 |
|  | AG | 0.046 | 0.039 | 0.051 | 0.232 |
|  | GA | 0.042 | 0.051 | 0.036 | 0.107 |
| TNFRSF1A^*^ | TCA | 0.606 | 0.610 | 0.604 | 0.804 |
|  | TTA | 0.249 | 0.240 | 0.254 | 0.480 |
|  | CTG | 0.096 | 0.093 | 0.097 | 0.737 |
|  | CTA | 0.023 | 0.019 | 0.025 | 0.393 |
|  | TTG | 0.010 | 0.016 | 0.006 | 0.049^a^ |
| TNFRSF1B^&^ | TG | 0.468 | 0.465 | 0.470 | 0.835 |
|  | TA | 0.354 | 0.358 | 0.351 | 0.781 |
|  | GG | 0.104 | 0.099 | 0.107 | 0.602 |
|  | GA | 0.074 | 0.078 | 0.072 | 0.621 |

^#^ The order of SNPs in TNF-α is rs1800629 and rs361525.

^*^The order of SNPs in TNFRSF1A is rs767455, rs4149577 and rs1800693.

^&^The order of SNPs in TNFRSF1A is rs1061622 and rs1061624.

^a^P=0.130 after correction for multiple testing
