# Supplementary material for: Association of TNF-α, TNFRSF1A and TNFRSF1B Gene Polymorphisms with the Risk of Sporadic Breast Cancer in Northeast Chinese Han Women
Source: PLoS One. 2014 Jul 10;9(7):e101138. doi: 10.1371/journal.pone.0101138 (PMC4091942; doi:10.1371/journal.pone.0101138)
Supplement: Table S11 — Genotyping information for TNF-α, TNFRSF1A and TNFRSF1B SNPs. (DOC) [file pone.0101138.s012.doc]

Table S11 Genotyping information for TNF-α, TNFRSF1A and TNFRSF1B SNPs

| SNP ID | Forward and reverse primers (5’-3’) | NEB cutter^1^ | Annealing  temperature | product  length |
| --- | --- | --- | --- | --- |
| TNF-α  rs1800629 | F 5’-AGGCAATAGGTTTTGAGGGCCAT-3’ | NcoI | 61.0°C | 201bp |
|  | R 5’-TGCACCTTCTGTCTCGGTTTCTT-3’ |  |  |  |
| rs361525 | F 5’-AGAAGACCCCCCTCGGAACC -3’ | MspI | 61.0°C | 219bp |
|  | R 5’-AGAGGAGGGcGGGGAAAGAA-3’ |  |  |  |
| TNFRSF1A rs767455 | F 5’-AGTGGCTGAGGTTAGGAC-3’ | BsrI | 53.9°C | 330bp |
|  | R 5’-CTATGCCCGAGTCTCAAC-3’ |  |  |  |
| rs4149577 | F 5’-GCAAGTTAAAGCCTGAATGAAG-3’ | BspCNI | 56.0°C | 312bp |
|  | R 5’-ATGACCATTTCCCTGACCC-3’ |  |  |  |
| rs1800693 | F 5’-ACTGTGTTTCATTCTTCTGC-3’ | BstEII | 56°C | 444bp |
|  | R 5’-TAAACCAATGAAGAGGAGG-3’ |  |  |  |
| TNFRSF1B rs1061622 | F 5’-GCACACATCGTCACTCTC-3’ | NlaIII | 56.2°C | 379bp |
|  | R 5’-AAGGAGTGAATGAATGAGAC-3’ |  |  |  |
| rs1061624 | F 5’-CTGTGTCGTAGCCAAGGTG-3’ | MspA1I | 57.4°C | 278bp |
|  | R 5’-GGCAGGTCACAGAGAGTCAG-3’ |  |  |  |

^1^NEB Cutter, names of restriction enzymes purchased from the NEB biolabs company.
